# A new measurement of sequence conservation

**DOI:** 10.1186/1471-2164-10-623

**Published:** 2009-12-22

**Authors:** Xiaohui Cai, Haiyan Hu, Xiaoman Li

**Affiliations:** 1 Center for Research in Biological Systems, University of California, San Diego, 9500 Gilman Dr. MC0446, La Jolla, CA 92093, USA; 2 School of Electrical Engineering and Computer Science, University of Central Florida, 4000 Central Florida Blvd, Orlando, FL, 32816, USA; 3 Burnett School of Biomedical Science, College of Medicine, University of Central Florida, 4000 Central Florida Blvd, Orlando, FL, 32816, USA

## Abstract

**Background:**

Understanding sequence conservation is important for the study of sequence evolution and for the identification of functional regions of the genome. Current studies often measure sequence conservation based on every position in contiguous regions. Therefore, a large number of functional regions that contain conserved segments separated by relatively long divergent segments are ignored. Our goal in this paper is to define a new measurement of sequence conservation such that both contiguously conserved regions and discontiguously conserved regions can be detected based on this new measurement. Here and in the following, conserved regions are those regions that share similarity higher than a pre-specified similarity threshold with their homologous regions in other species. That is, conserved regions are good candidates of functional regions and may not be always functional. Moreover, conserved regions may contain long and divergent segments.

**Results:**

To identify both discontiguously and contiguously conserved regions, we proposed a new measurement of sequence conservation, which measures sequence similarity based only on the conserved segments within the regions. By defining conserved segments using the local alignment tool CHAOS, under the new measurement, we analyzed the conservation of 1642 experimentally verified human functional non-coding regions in the mouse genome. We found that the conservation in at least 11% of these functional regions could be missed by the current conservation analysis methods. We also found that 72% of the mouse homologous regions identified based on the new measurement are more similar to the human functional sequences than the aligned mouse sequences from the UCSC genome browser. We further compared BLAST and discontiguous MegaBLAST with our method. We found that our method picks up many more conserved segments than BLAST and discontiguous MegaBLAST in these regions.

**Conclusions:**

It is critical to have a new measurement of sequence conservation that is based only on the conserved segments in one region. Such a new measurement can aid the identification of better local "orthologous" regions. It will also shed light on the identification of new types of conserved functional regions in vertebrate genomes [1].

## Background

The identification of the conserved regions of a genome is fundamentally important. The importance lies in the fact that conserved regions are often functional. For instance, many studies have shown that conserved regions correspond to coding genes, non-coding RNAs, enhancers, and other functional regions [2-4]. With many regions in the human genome that are largely of unknown function, the identification of the conserved regions is critical to accelerate the process of understanding the function of the human genome. Note that, in this paper, conserved regions are the regions that share at least a certain degree of sequence similarity with their homologous regions in other species [2,3]. Therefore, conserved regions are good candidates of functional regions and may not be functional regions sometimes. Moreover, different from previous studies, conserved regions may contain long and divergent segments.

There are many methods available for the identification of conserved regions. Early methods require conserved human regions to be at least 70% identical over at least 100 base pairs (bps) long ungapped alignment of human and mouse sequences [2,3]. Later, methods that are more sophisticated have been developed with given pairwise or multiple sequence alignments to define conservation regions [4-6]. All of these methods are based on contiguous sequence similarity between or among aligned sequences, which requires that the contiguous regions under study are similar to the aligned regions in other species in order to be claimed as conserved regions. There may be a few short divergent segments in such conserved regions. However, the overall sequence similarity of the conserved regions compared with their aligned regions still needs to be high. Here and in the following, the overall sequence similarity is defined as the percentage of aligned identical nucleotides in the alignments of the entire region. Note that these methods identify conserved regions from pre-aligned sequences, which makes them vulnerable to the quality of the pre-aligned sequences [7].

**Figure 1:**
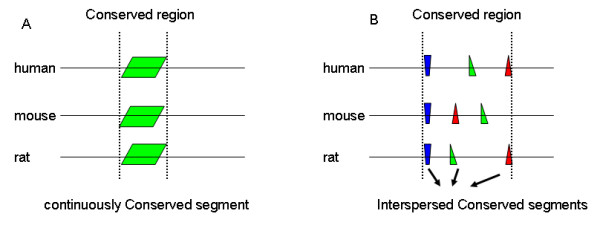
**The two types of conserved regions**. The three horizontal lines represent the orthologous sequences in human, mouse and rat. The small boxes with the same color on each line represent the corresponding conserved segments in different species. The regions between two dotted lines are the conserved regions. (A). A contiguously conserved region. (B) A conserved region with interspersed conserved segments.

Besides the above contiguously conserved regions that can be identified by available methods, there are a large number of conserved regions with Interspersed Conserved Segments (ICS) that cannot be identified by the above methods (Figure 1). The number of conserved regions with ICS is enormous in vertebrate genomes [8]. A well known example is the conserved *cis*-regulatory modules (CRMs) [9,10]. A CRM may be highly similar to its orthologous CRM only around a few transcription factor binding sites (TFBSs). This is because a CRM is a short DNA sequence that contains multiple TFBSs and maybe only these TFBSs are functional and conserved. Shashikant et al have shown such a pair of experimentally verified functional CRMs in Hoxc8, which function in both mouse and fugu, and are composed of several short conserved segments separated by divergent sequences [11]. Obviously, due to the discontiguous sequence conservation and the low overall sequence similarity (50.5% identities in the case of the CRM in Hoxc8), in spite of their functional conservation, such regions with ICS may not be aligned well by any single alignment and will be missed by the above conservation studies.

Besides CRMs, many DNase I hypersensitive regions are shown below to contain ICS. There could be new types of functional regions with ICS as well. Note that in such conserved regions, it is possible that all the ICS work together to perform a function. The above methods either consider individual ICS separately, or neglect them. Therefore, such conserved regions with ICS are missed completely or partially, because any individual ICS may be not statistically significant. It is thus critical to have a new measurement of sequence conservation that considers the conservation of all ICS in a region simultaneously.

To identify both types of conserved non-coding regions, we proposed a new measurement of sequence conservation. Under this measurement, the sequence similarity is calculated by using only the conserved segments in the regions under consideration (see Methods). Therefore, two orthologous regions with a low overall sequence similarity could be detected as conserved. By developing a local alignment-based procedure with the new measurement, we analyzed the conservation of 1642 human functional regions from the ENCyclopedia Of DNA Elements (ENCODE) project [12] in the mouse genome (see Methods). These 1642 functional regions include 172 regions defined from chromatin immunoprecipitation followed by microarray experiments (TFBS-clustered regions) and 1470 regions defined by DNase I hypersensitivity-related experiments (DHS regions).

We found that there are two or more ICS in 70.3% of TFBS-clustered regions and 17.0% of DHS regions. Moreover, at least 11% of human functional regions that contain multiple ICS would be neglected based on contiguous sequence similarity. We also noticed that for more than 72.9% of the 1642 human regions, our procedure identifies mouse regions that are more similar to the human regions than those mouse regions aligned in the UCSC (University of California, Santa Cruz) genome browser [13]. We also compared the homologous regions obtained from our procedure with those obtained from BLAST [14] and MegaBLAST [15]. We found that the mouse regions identified from our procedure comprise the best BLAST and MegaBLAST hits for all functional regions with significant hits (the smallest E-value less than 1E-10). However, BLAST and MegaBLAST missed several conserved segments in more than 29.3% regions and our procedure identifies all of the BLAST/MegaBLAST hits in all of the regions. Our observation from the study of the conservation of these functional regions may change the way people define sequence conservation and may shed light on the identification of new types of functional regions.

## Results

Our new measurement of sequence conservation calculates the sequence similarity based on conserved segments. To obtain the conserved segments, we apply the local alignment software CHAOS [16] to a pair of human-mouse orthologous non-coding sequences (see Methods for details). The aligned human-mouse segments outputted from CHAOS are called conserved segments. Note that these conserved segments may not be in the same order as that in the input human-mouse sequences (Figure 1(B)). We use CHAOS instead of other local alignment software because CHAOS has been shown to correctly align regulatory elements in distant species [16] in long sequences. We do not use global alignment methods to define conserved segments because a recent study has shown that three most popular methods cannot align a portion of coding regions consistently [7]. Moreover, conserved regions with ICS are difficult to align well by one single alignment.

With this definition of conserved segments, we implement the following three-step procedure to calculate the conservation score for an *m*-kilobase (kb) long human region. Assume this human region is in the non-coding region of the gene H1. The ortholog of H1 in the mouse genome is M1. First, we apply the CHAOS software to identify conserved segments in the non-coding region of M1, by comparing this *m*-kb long human region with the non-coding region of M1. Here and in the whole paper, the non-coding sequence of a gene includes the upstream sequences until the closer endpoint of the 5' adjacent gene, the intron sequences of this gene, and the downstream sequences until the closer endpoint of the 3' adjacent gene. Depending on which codon is closer to this gene under consideration, the endpoint could be the start codon or the stop codon of the adjacent genes. Second, for any *m*-kb long mouse region starting from a conserved mouse segment, we calculate the score of the mouse region and the *m-*kb long human region, by summing the scores of the aligned conserved segments within this pair of *m*-kb long regions. We obtain the score of a pair of aligned conserved segments from the CHAOS output. Third, we define the conservation score of the *m*-kb long human region as the best score obtained at the second step. The corresponding *m*-kb long mouse region with the best score is claimed as the mouse homologous region of the human region. If the above H1 has multiple mouse orthologs, we will use the non-coding regions of all of the orthologs to carry out the above three-step procedure.

We applied the above procedure to the 172 TFBS-clustered regions and 1470 DHS regions. For each human region, we identified the best mouse homologous region. In the following, we described our observations regarding these homologous regions and compared these regions with the homologous regions defined by the UCSC genome browser and those defined by BLAST and MegaBLAST [15].

### More than 17.0% human functional regions contain ICS

**Figure 2:**
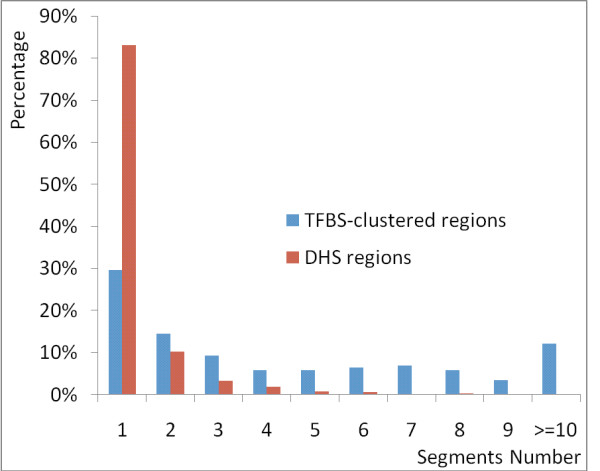
**The number of conserved segments in the human functional regions**. The x-axis is the number of conserved segments. The y-axis is the percentage of the regions that contain the corresponding number of conserved segments. Here the percentage refers to the number of the regions instead of the length of the regions. As was shown in the figure, in a large number of human functional regions, there are two or more ICS separated by divergent sequences.

We found that many human functional regions contain ICS instead of contiguously conserved sequences. In 121 (70.3%) TFBS-clustered functional regions and 250 (17.0%) DHS functional regions, there are two or more ICS that are separated by divergent sequences (Figure 2). In 112 of the 121 (92.6%) TFBS-clustered regions and in 162 of the 250 (64.8%) DHS regions, the overall sequence similarity between the human functional region and the corresponding mouse homologous region is less than 70%. Note that the homologous regions based on the new measurement often comprise the best mouse BLAST hits and the best MegaBLAST hits when using the corresponding human regions as queries against the mouse sequences, which shows that the homologous regions are most likely orthologous [14]. It is thus clear that the current conservation measurement that requires contiguous sequence similarity may claim that 11% (17%*64.8%) of these functional regions are not conserved. That is, without a new measurement of sequence conservation, at least 11% of the conserved functional regions would be missed by the current methods.

**Figure 3:**
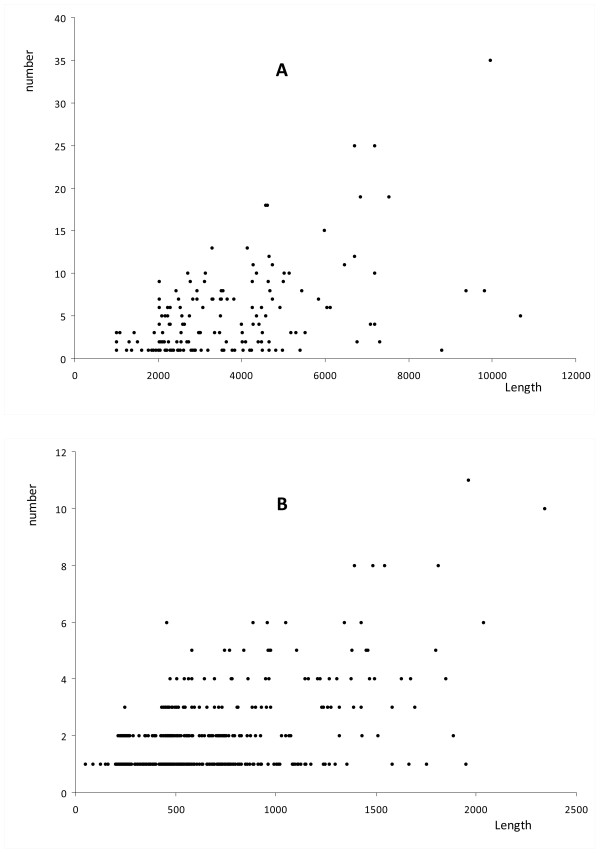
**The length versus the number of conserved segments in a region**. The x-axis is the length of the functional regions and y-axis is the number of conserved segments found in these regions. (A) TFBS-clustered functional regions. (B) DNase I Hypersensitivity regions, respectively.

To see what may contribute to the number of ICS in functional regions, we checked whether the number of ICS in a functional region is determined by the length of the functional region. First, for the TFBS-clustered regions, the length of the region has a low correlation coefficient, 0.516, with the number of the ICS in the region. Moreover, the longest regions do not have the largest numbers of the ICS (Figure 3). This may suggest that the ICS represent the intrinsic functional sequences while the sequences between the adjacent ICS represent the non-functional sequences. Second, for the DHS regions, it is clear that length of a region does not determine the number of ICS in the region. For the DHS regions, the average length and the median length of the regions is 393 bp and 251 bp, respectively, which is similar to the size of many known CRMs. However, we found that 250 (17.0%) DHS regions still contain two or more ICS. Moreover, 26.4% of the DHS regions containing two or more ICSs are shorter than the average length of the DHS regions. Note that, by chance, it is common for a long region of a few thousand bps to contain several ICS and it is not usual for a short sequence of 393 bp to contain two or more ICS. Thus, the observation from the DHS regions clearly indicates that the ICS may be functional segments in these regions. Because these 26.4% of the DHS regions are short, most likely these regions are functional units and the conserved segments in each of these units work together to perform a function.

**Figure 4:**
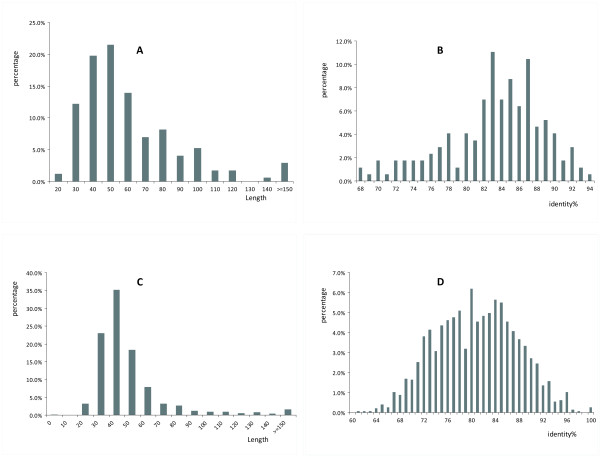
**The distribution of the length and the overall percentage of identities of the conserved segments**. (A) and (B) are for TFBS-clustered regions. (C) and (D) are for DHS regions.

We next analyzed the length and the conservation of ICS in the functional regions (Figure 4). We noticed that, in 57 (33.1%) TFBS-clustered regions and in 905 (61.6%) DHS regions, the average length of the ICS are less than 50 bp. Meanwhile, for the TFBS-clustered regions, the average similarity of the ICS compared with their aligned counterpart, defined as the percentage of the identities based on the CHAOS alignments, is 83.7%, with 68.1% as the minimum and 94.1% as the maximum. For the DHS regions, the average similarity of the ICS compared with their orthologous counterpart, is 81.3%, with 61.5% as the minimum and 100% as the maximum. From the length and the conservation of the ICS in functional regions (Figure 4), it is clear that the length distributions of the ICS in TFBS-clustered regions and in DHS regions are on the same scale. That is, the length of the ICS is not related to the length of the whole functional regions, which partially agrees with the observation that the number of the ICS in a region does not depend on the length of the regions.

We also investigated whether these discontiguously conserved regions are biologically meaningful. We scanned the discontiguously conserved regions using the known motifs in the TRANSFAC database and using stringent score cutoff to define TFBSs (p-value < 0.0001). We found that ICS in both the TFBS-clustered regions and the DHS regions contain conserved TFBSs. On the other hand, we did not find conserved TFBSs in the sequences between adjacent ICS in these regions. For instance, we found seven conserved TFBSs in the two ICS in the DHS region id-1244 (chr11:130824648-130824895). In another example, we found more than 3 TFBSs on average in each of the seven ICS in the TFBS-clustered region id-211591 (chr1:149712431-49714909). These putative TFBSs in the ICS support that these ICS may be responsible for the function of these regions.

### Our procedure provides mouse homologous regions that are more similar to the human regions

From the above analyses, we already know that there exist a large number of conserved regions with ICS. Here we show that our procedure provides mouse homologous sequences that are more similar to the human functional sequences compared with the aligned mouse sequences from genome alignments, another advantage of the new measurement.

**Figure 5:**
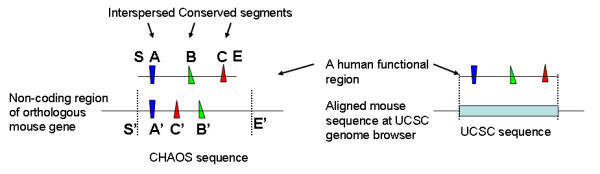
**A CHAOS sequence and the corresponding UCSC sequence**. The two sequences between the two pairs of dotted lines are the CHAOS sequence and the UCSC sequence for the same human functional region. In more detail, in the left panel, assume S and E are the start position and the end position of the human region; A, B, C are the conserved human segments and A', B', C' are the corresponding mouse segments; the distance between S' and the left end of the segment A' is equal to the distance between S and the left end of the segment A; the distance between E' and the left end of the segment B' is equal to the distance between E and the left end of the segment B. Then the mouse sequence between S'E' is the CHAOS sequence.

For every human functional region, we obtained the mouse homologous regions as above. In every mouse homologous region, we defined a CHAOS sequence as the sequence comprised of the conserved segments and other sequences at both sides to cover the entire human region. For example, the CHAOS sequence in Figure 5 is the mouse sequence from the location S' to the location E'. We also obtained the corresponding mouse region from the UCSC genome browser, which is aligned with exactly the entire human region. We call these mouse sequences UCSC sequences (Figure 5). To measure the similarity of the CHAOS sequences and the corresponding human sequences, we use LAGAN [17] to align the CHAOS sequences with the human sequences. For the UCSC sequences, we use the alignments obtained from the UCSC genome browser. We define the similarity as the percentage of aligned identical nucleotides in the alignments.

We found that CHAOS sequences are often more similar to the corresponding human sequences than the UCSC sequences are to the human sequences. In 139 out of 172 (80.8%) TFBS-clustered regions and in 842 out of 1470 (57.3%) DHS regions, the CHAOS sequences are more similar to the corresponding human sequences than the UCSC sequences. For these 139 TFBS-cluster regions and 842 DHS regions, the CHAOS sequences have on average 22.2% and 29.9% more identities than the UCSC sequences, respectively. This shows that the new measurement may be a better way to measure the similarity of orthologous region for non-coding sequences. It also implies that the current conservation studies may have missed many conserved regions by calculating conservation scores based on genome alignments.

Besides the above 139 TFBS-clustered regions and 842 DHS regions, we found 2 TFBS-clustered regions and 61 DHS regions for which the UCSC sequences are as similar to the human sequences as the CHAOS sequences. Moreover, in 31 (18.0%) TFBS-clustered regions and 567 (38.6%) DHS regions, the UCSC sequences are more similar to the corresponding human sequences. Note that the CHAOS sequences may be not so similar to the human sequences as the UCSC sequences, since the CHAOS sequences are identified based on the conserved segments only. In the following, we wanted to investigate whether this was the case.

**Figure 6:**
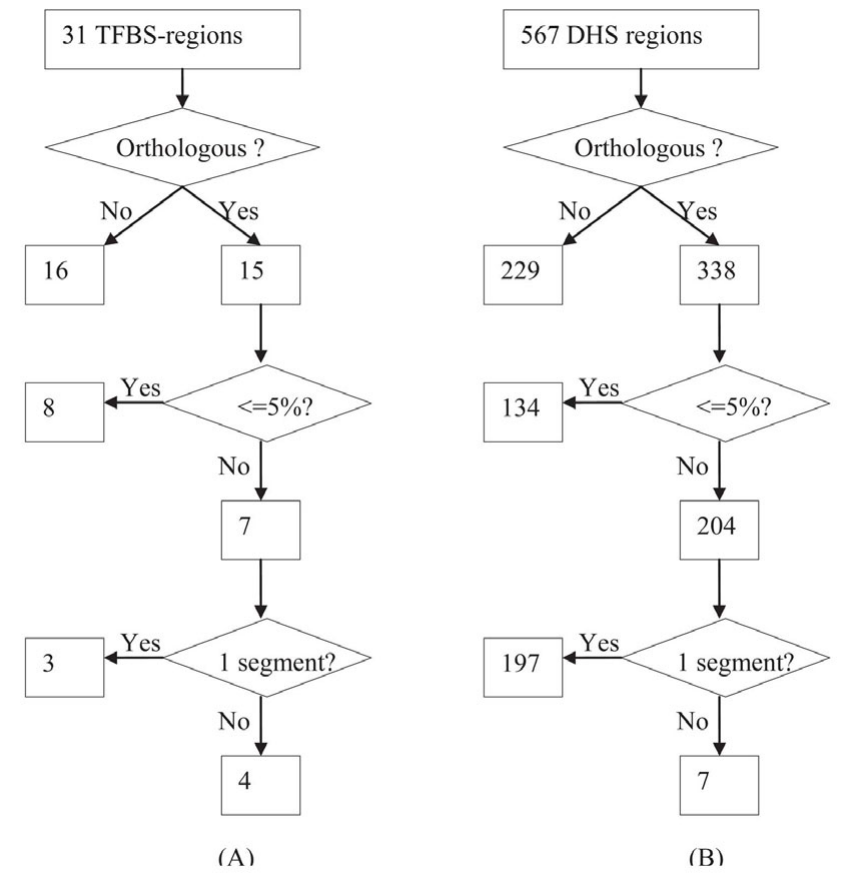
**Four types of functional regions where the UCSC sequences have a higher overall similarity to the human sequences than the CHAOS sequences**.

We found that there were four types of functional regions where the UCSC sequence was more similar to the human sequence (Figure 6). First, for 16 TFBS-clustered regions and 229 DHS regions, the UCSC sequences were not from non-coding regions of the orthologous genes. Thus, for these regions, the CHAOS sequences are in fact better than the UCSC sequence to measure the conservation. Second, in 8 of the remaining 15 TFBS-clustered regions and in 134 of the remaining 338 DHS regions, the difference between the percentages of identities in UCSC sequences and in CHAOS sequences is less than 5%. Such small differences are mostly due to the different end positions in the genome. Third, for the remaining 7 TFBS-clustered regions and the remaining 204 DHS regions, 3 TFBS-clustered regions and 197 DHS regions have only one conserved segment from the CHAOS software. These individual conserved segments have 15% higher identity than the UCSC sequences compared with the human regions, which shows that the UCSC sequences may be misleading by misaligning the most highly conserved sub-regions in functional regions. Fourth, for the remaining 4 TFBS-clustered regions and 7 DHS regions, the UCSC sequences and the CHAOS sequences do overlap more than 80%. The difference is caused by the repeats and exons. Note that the CHAOS sequences do not include repeats and exons while the UCSC sequences can include them. When the corresponding CHAOS sequences only match part of the human regions while the UCSC sequences can match the whole human regions with repeats and exons, the UCSC sequences will have better overall similarity than the CHAOS sequences although they overlap significantly.

In summary, in at least 90.1% (139+16 out of 172) TFBS-clustered regions and 72.9% (842+229 out of 1470) DHS regions, the CHAOS sequences are more similar to the human sequences than the UCSC sequences are to the human sequences, in terms of percent identity in the sequence alignments. Such a dominant performance from the new measurement confirms that genome alignments based on contiguous sequence similarity may misalign many conserved regions. For the remaining regions, although the UCSC sequences are the same or more similar to the human sequence, they often misaligned the most conserved sub-regions. Thus, it is questionable that UCSC sequences provide better counterparts in the remaining regions.

### Genome alignments misaligned many functional regions

In the previous section, we have shown that many UCSC sequences are not from the non-coding regions of the orthologous genes. We have also shown that the UCSC sequences are not as similar to the human sequences as the CHAOS sequences in most regions. Moreover, in the regions where the UCSC sequences are more similar, we found that the most conserved segments in the UCSC sequences may be misaligned. We found two factors can contribute to this.

First, the genome alignment considers contiguous sequence similarity, which makes it difficult to align some local regions. For instance, due to genome rearrangements during evolution, some parts of a functional region are kept in the original 5'-3' direction while other parts are inverted to 3'-5' directions. Thus, the overall sequence similarity based on genome alignments for true orthologous regions is too low to be identified. Therefore, genome alignments may poorly align these regions across species. For instance, the DHS region id-2404 (chr16:61153038-61153304) shares 75.4% identities with the CHAOS sequence (chr8:102219277-102219500) and shares 49.8% identities with the UCSC sequence (chr8:102870981-102871214). The much lower percent identity from the UCSC sequence is due to the fact that the segment (chr16:61153109-61153157) and the segment (chr16:61153242-61153294) in this DHS region are inverted in the mouse genome. In the CHAOS sequence, the two segments are aligned with two segments chr8+:102219348-102219395 and chr8-:102219440-102219395, which occur in the positive strand and negative strand, respectively ("+" and "-" following the chromosome name mean the positive and negative strand, respectively). In the UCSC sequence, the two segments are aligned with two segments, chr8+:102871048-102871095 and chr8+:102871168-102871203, in the positive strand.

Second, the genome alignments are targeting genome scale sequence similarity and thus may sacrifice the alignment quality of short functional regions. For instance, for the DHS region id-5225 (chr5	:142205165-142205746), we found that there is a conserved segment of 101 bp long with 83% identity to its orthologous region in the mouse genome. The genome alignment at UCSC aligned this region with all gaps. It is clear that, to provide better genome scale matches, the genome browser cannot guarantee to align the corresponding sequences for short regions.

### BLAST and MegaBLAST neglect many conserved segments

The comparison of the CHAOS sequences with the UCSC sequences in previous sections shows that the aligned sequences from the UCSC genome browser may be misleading when considering the evolution of a local region. Because we are trying to identify the most similar regions around an orthologous mouse gene for a human query sequence, it is also necessary to determine the difference between our approach and BLAST, the basic tool for the same purpose using contiguous sequence similarity [14].

For the above 172 TFBS-clustered regions and the 1470 DHS regions, we applied BLAST with the default parameters to output hits from the non-coding regions of the orthologous mouse genes. Note that the orthologous mouse genes were obtained from the Mouse Genome Informatics database (MGI) [18]. We found that the best BLAST hits in the mouse were always included in the corresponding CHAOS sequences, for all human regions with significant BLAST hits (the smallest E-value < 1E-10; see Table 1 for details). The inclusion of the best BLAST hits in the CHAOS sequences supports that our procedure identified reliable "orthologous" regions when the query sequences were "conserved".

**Table 1:** The comparison of the CHAOS sequences with the BLAST hits.

#regions	#TFBS-clustered regions (172)	#DHS regions (1470)
E-value < 1	165	1464

E-value < 1E-10	76	270

E-value < 1 & Overlap	122	675

E-value < 1 & Non-overlap	43	789

E-value < 1E-10 & Overlap	76	270

E-value < 1E-10 & Non-overlap	0	0

"Overlap" means the best BLAST hits are included in the corresponding CHAOS sequences. "Non-overlap" means the best BLAST hits do not overlap with the corresponding CHAOS sequences.

To show the benefit of measuring the sequence similarity based on conserved segments without considering divergent sequences in a region, we further examined the human regions with significant mouse BLAST hits. We found that in 71 out of 76 (93.4%) TFBS-clustered regions and 167 out of 270 (61.9%) DHS regions, there were one or more CHAOS segments that were missed by BLAST (Table 1). In the remaining regions, the CHAOS segments had a one-to-one correspondence with the BLAST hits, including the hits with E-values larger than 1E-10. Note that the human query sequences are experimentally verified to be functional. It is most likely that all the ICS in such regions, especially in the short DHS regions, are working together to perform functions. Therefore, BLAST missed many ICS by considering conserved segments individually. It also implies that many significantly conserved regions could be missed by BLAST if there is no individual significant hit. On the other hand, the identified ICS in a region together may tell us new functions of the region.

We also applied discontinuous MegaBLAST [15] to identify mouse homologous regions for these human functional regions. Discontinuous MegaBLAST is claimed to be able to identify more divergent sequence similarity than BLAST. By using a recommended parameter combination "-A 50 -t 21 -W 11 -N 1", we can only identify hits in 94 out of 172 TFBS-clustered regions and in 306 out of 1470 DHS regions (Table 2). Among them, only 76 TFBS-clustered regions and 246 DHS regions have MegaBLAST hits with an E-value less than 1E-10. In all these regions, our procedure identified all MegaBLAST hits as conserved segments. In 71 out of the 76 (93.4%) TFBS-clustered regions and in 72 out of the 246 (29.3%) DHS regions, our procedure identified more conserved segments than MegaBLAST. Although the results may be affected by the default parameters we used, the fact that MegaBLAST missed conserved segments in so many regions shows that MegaBLAST cannot identify many conserved regions with ICS.

**Table 2:** The comparison of the CHAOS sequences with the discontiguous MegaBLAST hits.

#regions	#TFBS-clustered regions (172)	#DHS regions (1470)
E-value < 1	94	306

E-value < 1E-10	76	246

E-value < 1 & Overlap	91	280

E-value < 1 & Non-overlap	3	26

E-value < 1E-10 &overlap	76	246

E-value < 1E-10 & non-overlap	0	0

"Overlap" means the best MegaBLAST hits are included in the corresponding CHAOS sequences. "Non-overlap" means the best MegaBLAST hits do not overlap with the corresponding CHAOS sequences.

### At least 12.8% human functional regions are conserved in mouse

In the previous sections, we have shown that it is necessary to extend the current conservation measurements to consider only the conserved segments in a region. Here we want to estimate the percentage of human functional regions conserved in the mouse based on our new conservation measurement and the functional regions mentioned above.

**Figure 7:**
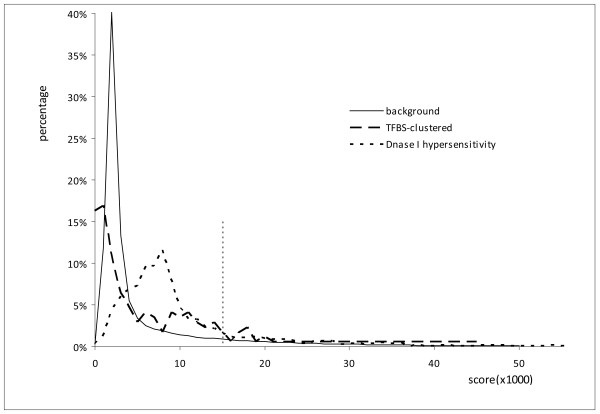
**The distributions of the conservation scores**. The distribution of the conservation score of a random 1 kb long human non-coding region aligned with mouse sequence is plotted with a solid line. The distributions of the conservation score of functional human regions are plotted in dashed lines.

To determine whether a human region is conserved in mouse (see Methods for detail), we first calculated how conserved a random human non-coding region is in the mouse genome. Figure 7 shows the distribution of the conservation score of a random 1 kb long human non-coding repeat-free sequence. Note that this conservation score is a sum of the similarity scores of individual CHAOS segments in a pair of 1 kb long regions. Since it is estimated that at least 3.5% of human non-coding sequences are under constraint [19], we choose the top 3.5% cut-off in this distribution to define the conserved regions. Note that we assume the constrained sequences are conserved here, which may not be true for some short constrained sequences. Please also keep in mind that, the 3.5% may be an underestimate of the constraint sequences in the human genome [20]. For any region with a conservation score in the top 3.5% of scores for random regions, we claim this region is conserved between human and mouse. We next calculated the conservation score of a functional human region. By comparing these two distributions, we found that about 12.8% of (22 out of 172) TFBS-clustered regions are conserved in mouse, and about 19.2% of (279 out of 1470) DHS regions are conserved in mouse.

If we consider the contiguous sequence similarity for the TFBS-clustered conserved regions, the percentage of sequence identity is from 46.1% to 78.0%, with a median of 67.4%. For the DHS conserved regions, the percentage of sequence identity is from 23.8% to 92.1%, with a median of 69.8%. It is thus evident that many conserved regions are neglected by the current conservation studies.

## Discussion

We proposed a new measurement of sequence conservation. Compared with current measurements based on contiguous sequence similarity in local or global alignments, this new measurement considers interspersed sequence similarity. Therefore, the conserved regions based on the new measurement will include the conserved regions defined by the existing methods. Moreover, the conserved regions based on the new measurement will also include the conserved regions with ICS that are missed by current measurements, such as some conserved CRMs [9,10] and many DHS regions.

The advantage of the new measurement over the current measurement is demonstrated in the functional regions we studied. First, many functional regions can be easily missed by the current conservation studies while they are identified by our method based on the new measurement. We found that 121 (70.3%) TFBS-clustered functional regions and 250 (17.0%) DHS functional regions contain two or more ICS. If we consider contiguous sequence similarity, 112 of the 121 (92.6%) TFBS-clustered regions and 162 of 250 (64.8%) DHS regions have an overall sequence identity of less than 70% compared with their homologous regions. Therefore, at least 11% (17%*64.8%) of regions containing multiple ICSs are neglected by the current conservation methods. Second, our procedure based on the new conservation measurement provides homologous regions that are more similar to the human regions than the aligned sequences in the genome alignments. Third, our procedure identifies a larger number of conserved segments in homologous regions than BLAST and MegaBLAST.

The new conservation measurement is similar to the normalized sequence similarity [21]. Both methods will normalize the sequence similarity by the sequence length. However, the normalized sequence similarity is aimed at identifying regions with percentage of identities larger than a pre-specified threshold. It is still considering every bp in a region to measure the sequence similarity. The new measurement considers only the conserved segments to calculate the sequence similarity.

It is understandable that conserved CRMs may only contain several ICS compared with their orthologous CRMs. We notice that many DHS regions shorter than 400 bp long also contain ICS, which shows that there may be functional regions other than CRMs that also share ICS with their counterparts. We thus need to adopt the new measurement of sequence conservation in order to have better understanding of conservation and to perform novel comparative genomics analyses.

The new measurement of sequence conservation proposed in this paper will significantly affect how people study evolution. Our study here shows that the classical measurement will miss 11% of conserved functional regions between human and mouse. This has two implications. First, there may be many more sequences conserved between human and mouse than we currently estimate, which is consistent with the argument in a recent paper [20]. Second, with more divergent species, the percentage of missed conserved regions by the classical measurement may be even larger, given the fact that orthologous sequences are more divergent and orthologous sequences contain more ICS [7].

Note that the conserved functional regions defined above may not be functional in mouse. Although a functional human region shares ICS with a mouse region and the conservation is significant compared with that of random sequences, the function of the mouse region needs to be experimentally verified. Moreover, in this study, we implemented a procedure based on the local alignment software CHAOS, which may still miss some conserved segment candidates. Future studies independent of alignments should detect even more conserved regions. With the verification of the function of these mouse regions and further improvements of the method, we may finally estimate how many conserved regions are functional.

## Conclusions

We have proposed a new measurement of sequence conservation. By studying the human functional regions, we found that the new measurement is necessary since the functional regions with ICS are not rare and these regions are not considered as conserved regions under the current measurement. Moreover, for most human regions under study, the homologous mouse regions identified under the new measurement have better overall sequence similarities to the human regions than the corresponding regions identified using the current measurements. That is, there could be many conserved regions missed by using the current measurement. Thus, to apply the new measurement to identify conserved regions and to understand the function of the ICS in the conserved regions may change the way people study comparative genomics and may enable the identification of new types of functional elements.

## Methods

### Collection of functional regions

We collected two sets of functional regions. The first set contained 689 TFBS-clustered functional regions based on chromatin immunoprecipitation followed by microarray experiments for 29 transcription factors [22]. The second set [23] contained 8217 DHS regions based on quantitative chromatin profiling [24], massively parallel signature sequencing [25] and DNase-chip [26]. Both types of functional regions are the non-coding regions from the published results of the ENCODE project [22,23].

We further selected the functional regions based on two criteria. First, the functional regions fell into the 30 random regions selected by the ENCODE project. We did not use the functional regions from the other 14 manually selected ENCODE regions in order to draw more unbiased conclusions. Second, the functional regions fell into the non-coding regions of the 13628 human refseq genes that have mouse orthologs defined in the MGI database [18]. We did not consider rat orthologs because there are only 6991 human genes with rat orthologs in MGI. Certainly, our method can be easily extended to multiple species, in the similar way as extending pairwise alignments to multiple alignments (the conservation score will be defined as the sum of pairwise conservation score). In this manner, we obtained 172 TFBS-clustered functional regions and 1470 DHS functional regions in the human genome. The start positions, the end positions, and the original ID number of these regions are listed in additional files 1 and 22.

### Conservation score

We downloaded the human and mouse genome sequences from the UCSC genome browser website (version hg18 and mm8). The repeats in these sequences are already masked with lowercase alphabets. To define the conservation score, C(R), of a human region R of *m*-kb long, we implemented the three-step procedure below. For simplicity, assume R is in the non-coding region of the human gene H1. The mouse ortholog of H1 is M1 at MGI. Then , where nc(M1) is the non-coding region of M1 and R' is one m-kb long region in nc(M1), and S(R, R') is the sum of local alignment scores of all pairs of aligned segments in R and R' output from CHAOS [16]. CHAOS is a local alignment program for pairwise alignments. The basic idea of CHAOS is to identify similar k-mers (DNA segments of k bp) shared by two sequences and then to extend these k-mers to generate local alignments [16]. We used CHAOS as the local alignment tool because CHAOS is able to correctly align regulatory elements in distant species [16]. The details of the calculation of the C(R) are in the following sections.

First, we identified the conserved segments by using CHAOS to align the human region R with the non-coding sequences of M1. Here the non-coding sequence includes the upstream sequences, introns and downstream sequences of M1. The upstream sequence of M1 is the sequence from the closer endpoint of the 5' adjacent gene of M1 to the start codon of M1. The downstream sequence of M1 is the sequence from the stop codon of M1 to the closer endpoint the 3' adjacent gene of M1. Here the endpoint is either the start codon or the stop codon of the adjacent genes, depending on the orientation of the adjacent genes. When applying CHAOS, we set the word length parameter k as 6 bp, the number of degeneracy parameter as 0, and other parameters as default values in CHAOS. Since CHAOS can identify any significant local matches in two sequences, by setting the parameters in CHAOS in this way, we expected that multiple corresponding pairs of functional segments within a region in the two species would be aligned in some local alignments. Certainly, a long conserved region under the current conservation measurement will also be aligned by CHAOS. We call these aligned segments output from CHAOS conserved segments.

Second, we calculated the sequence similarity of R and every *m*-kb long mouse region that starts from a conserved segment in the non-coding region of M1. The similarity was defined as the sum of the scores of the local alignments of the conserved segments within this pair of *m*-kb long regions. Note that the local alignments and the score of local alignments were provided by CHAOS.

Third, we calculated the conservation score of R. The conservation score is defined as the best sequence similarity of R compared with an *m*-kb long mouse region R' in the non-coding sequence of M1, divided by *m*. Therefore, a long contiguously conserved region would have a high conservation score. On the other hand, some short regions with ICS will also have high score. Note that it takes O(nlogn) time to identify the best sequence similarity and the corresponding m-kb long mouse region R', if there are n mouse segments aligned with the human region R in the CHAOS output. For the 13628 human-mouse gene pairs we used, n is in the range of 0 to 259276 for m = 1.

### Conserved functional regions

In order to define the conserved functional regions, we generated the distribution of the conservation score of a random human region. We obtained this distribution by calculating the conservation score of every 1 kb long human non-coding region that starts with an aligned CHAOS segment in the local alignments of non-coding sequences of orthologous human-mouse genes.

With this background distribution of the conservation score, we defined a functional human region as a conserved functional region if the conservation score of this region is within the top 3.5% of the background distribution. We used 3.5% as a cutoff, because it is estimated that 3.5% of the non-coding sequences in the human genome are under constraint [19] and we assumed constraint sequences should be conserved. This assumption could be incorrect, but the analysis here should give a rough estimate of conserved regions.

## Authors' contributions

HH and XL designed the research. XC collected the data and analyzed the data. XC, HH and XL wrote the manuscript. All authors read and approved the final manuscript.

## Supplementary Material

Additional file 1 **172 TFBS-clustered regions used**. this file provides the genome coordinates of the 172 TFBS-clustered regions used in this paper. Click here for file

Additional file 2 **1470 DHS regions**. this file provides the genome coordinates of the 1470 TFBS-clustered regions used in this paper. Click here for file

## References

[B1] Chen X Zheng J Fu Z Nan P Zhong Y Lonardi S Jiang T Assignment of orthologous genes via genome rearrangement IEEE/ACM transactions on computational biology and bioinformatics/IEEE, ACM 2005 2 4 302 315 10.1109/TCBB.2005.48 17044168

[B2] Dermitzakis ET Reymond A Lyle R Scamuffa N Ucla C Deutsch S Stevenson BJ Flegel V Bucher P Jongeneel CV Numerous potentially functional but non-genic conserved sequences on human chromosome 21 Nature 2002 420 6915 578 582 10.1038/nature01251 12466853

[B3] Loots GG Locksley RM Blankespoor CM Wang ZE Miller W Rubin EM Frazer KA Identification of a coordinate regulator of interleukins 4, 13, and 5 by cross-species sequence comparisons Science (New York, NY) 2000 288 5463 136 140 10.1126/science.288.5463.136 10753117

[B4] Margulies EH Blanchette M Haussler D Green ED Identification and characterization of multi-species conserved sequences Genome research 2003 13 12 2507 2518 10.1101/gr.1602203 14656959 PMC403793

[B5] Siepel A Bejerano G Pedersen JS Hinrichs AS Hou M Rosenbloom K Clawson H Spieth J Hillier LW Richards S Evolutionarily conserved elements in vertebrate, insect, worm, and yeast genomes Genome research 2005 15 8 1034 1050 10.1101/gr.3715005 16024819 PMC1182216

[B6] Cooper GM Stone EA Asimenos G Green ED Batzoglou S Sidow A Distribution and intensity of constraint in mammalian genomic sequence Genome research 2005 15 7 901 913 10.1101/gr.3577405 15965027 PMC1172034

[B7] Margulies EH Cooper GM Asimenos G Thomas DJ Dewey CN Siepel A Birney E Keefe D Schwartz AS Hou M Analyses of deep mammalian sequence alignments and constraint predictions for 1% of the human genome Genome research 2007 17 6 760 774 10.1101/gr.6034307 17567995 PMC1891336

[B8] Davidson E The Regulatory Genome: Gene Regulatory Networks in Development and Evolution 2006 1 Burlington, MA: Academic Press

[B9] Arnone M Davidson EH The hardwiring of development: organization and function of genomic regulatory systems Development 1997 124 10 1851 1864 9169833 10.1242/dev.124.10.1851

[B10] Yuh C Bolouri H Davidson EH Genomic cis-regulatory logic: experimental and computational analysis of a sea urchin gene Science 1998 279 5358 1896 1902 10.1126/science.279.5358.1896 9506933

[B11] Shashikant CS Bolanowsky SA Anand S Anderson SM Comparison of diverged Hoxc8 early enhancer activities reveals modification of regulatory interactions at conserved cis-acting elements Journal of experimental zoology Part B 2007 308 3 242 249 10.1002/jez.b.21143 17171696

[B12] Birney E Stamatoyannopoulos JA Dutta A Guigo R Gingeras TR Margulies EH Weng Z Snyder M Dermitzakis ET Thurman RE Identification and analysis of functional elements in 1% of the human genome by the ENCODE pilot project Nature 2007 447 7146 799 816 10.1038/nature05874 17571346 PMC2212820

[B13] Kent WJ Sugnet CW Furey TS Roskin KM Pringle TH Zahler AM Haussler D The human genome browser at UCSC Genome research 2002 12 6 996 1006 12045153 10.1101/gr.229102 PMC186604

[B14] Altschul SF Gish W Miller W Myers EW Lipman DJ Basic local alignment search tool Journal of molecular biology 1990 215 3 403 410 2231712 10.1016/S0022-2836(05)80360-2

[B15] Ma B Tromp J Li M PatternHunter: faster and more sensitive homology search Bioinformatics (Oxford, England) 2002 18 3 440 445 10.1093/bioinformatics/18.3.440 11934743

[B16] Brudno M Chapman M Gottgens B Batzoglou S Morgenstern B Fast and sensitive multiple alignment of large genomic sequences BMC Bioinformatics 2003 4 66 10.1186/1471-2105-4-66 14693042 PMC521198

[B17] Brudno M Do CB Cooper GM Kim MF Davydov E Green ED Sidow A Batzoglou S LAGAN and Multi-LAGAN: efficient tools for large-scale multiple alignment of genomic DNA Genome research 2003 13 4 721 731 10.1101/gr.926603 12654723 PMC430158

[B18] Blake JA Eppig JT Richardson JE Bult CJ Kadin JA The Mouse Genome Database (MGD): integration nexus for the laboratory mouse Nucleic acids research 2001 29 1 91 94 10.1093/nar/29.1.91 11125058 PMC29788

[B19] Waterston RH Lindblad-Toh K Birney E Rogers J Abril JF Agarwal P Agarwala R Ainscough R Alexandersson M An P Initial sequencing and comparative analysis of the mouse genome Nature 2002 420 6915 520 562 10.1038/nature01262 12466850

[B20] Pheasant M Mattick JS Raising the estimate of functional human sequences Genome research 2007 17 9 1245 1253 10.1101/gr.6406307 17690206

[B21] Arslan AN EelO Pevzner PA A new approach to sequence comparison: normalized sequence alignment Bioinformatics 2001 17 4 327 323 10.1093/bioinformatics/17.4.327 11301301

[B22] Zhang ZD Paccanaro A Fu Y Weissman S Weng Z Chang J Snyder M Gerstein MB Statistical analysis of the genomic distribution and correlation of regulatory elements in the ENCODE regions Genome Res 2007 17 6 787 797 10.1101/gr.5573107 17567997 PMC1891338

[B23] King DC Taylor J Zhang Y Cheng Y Lawson HA Martin J Chiaromonte F Miller W Hardison RC Finding cis-regulatory elements using comparative genomics: some lessons from ENCODE data Genome Res 2007 17 6 775 786 10.1101/gr.5592107 17567996 PMC1891337

[B24] Sabo PJ Kuehn MS Thurman R Johnson BE Johnson EM Cao H Yu M Rosenzweig E Goldy J Haydock A Genome-scale mapping of DNase I sensitivity in vivo using tiling DNA microarrays Nat Methods 2006 3 7 511 518 10.1038/nmeth890 16791208

[B25] Crawford GE Holt IE Whittle J Webb BD Tai D Davis S Margulies EH Chen Y Bernat JA Ginsburg D Genome-wide mapping of DNase hypersensitive sites using massively parallel signature sequencing (MPSS) Genome Res 2006 16 1 123 131 10.1101/gr.4074106 16344561 PMC1356136

[B26] Crawford GE Davis S Scacheri PC Renaud G Halawi MJ Erdos MR Green R Meltzer PS Wolfsberg TG Collins FS DNase-chip: a high-resolution method to identify DNase I hypersensitive sites using tiled microarrays Nat Methods 2006 3 7 503 509 10.1038/nmeth888 16791207 PMC2698431

